# Brain perivascular spaces and autism: clinical and pathogenic implications from an innovative volumetric MRI study

**DOI:** 10.3389/fnins.2023.1205489

**Published:** 2023-06-23

**Authors:** Maria Alessandra Sotgiu, Alessandro Lo Jacono, Giuseppe Barisano, Laura Saderi, Vanna Cavassa, Andrea Montella, Paola Crivelli, Alessandra Carta, Stefano Sotgiu

**Affiliations:** ^1^Department of Biomedical Sciences, University of Sassari, Sassari, Italy; ^2^Unit of Child Neuropsychiatry, Department of Medicine, Surgery and Pharmacy, University of Sassari, Sassari, Italy; ^3^Department of Neurosurgery, Stanford University, Stanford, CA, United States; ^4^Clinical Epidemiology and Statistics Unit, Department of Medicine, Surgery and Pharmacy, University of Sassari, Sassari, Italy; ^5^Radiology Unit, Department of Medicine, Surgery and Pharmacy, University of Sassari, Sassari, Italy

**Keywords:** autism, glymphatic system, perivascular spaces, Virchow-Robin spaces, MRI

## Abstract

**Introduction:**

Our single-center case–control study aimed to evaluate the unclear glymphatic system alteration in autism spectrum disorder (ASD) through an innovative neuroimaging tool which allows to segment and quantify perivascular spaces in the white matter (WM-PVS) with filtering of non-structured noise and increase of the contrast-ratio between perivascular spaces and the surrounding parenchyma.

**Methods:**

Briefly, files of 65 ASD and 71 control patients were studied. We considered: ASD type, diagnosis and severity level and comorbidities (i.e., intellectual disability, attention-deficit hyperactivity disorder, epilepsy, sleep disturbances). We also examined diagnoses other than ASD and their associated comorbidities in the control group.

**Results:**

When males and females with ASD are included together, WM-PVS grade and WM-PVS volume do not significantly differ between the ASD group and the control group overall. We found, instead, that WM-PVS volume is significantly associated with male sex: males had higher WM-PVS volume compared to females (p = 0.01). WM-PVS dilation is also non-significantly associated with ASD severity and younger age (< 4 years). In ASD patients, higher WM-PVS volume was related with insomnia whereas no relation was found with epilepsy or IQ.

**Discussion:**

We concluded that WM-PVS dilation can be a neuroimaging feature of male ASD patients, particularly the youngest and most severe ones, which may rely on male-specific risk factors acting early during neurodevelopment, such as a transient excess of extra-axial CSF volume. Our findings can corroborate the well-known strong male epidemiological preponderance of autism worldwide.

## 1. Introduction

Autism spectrum disorder (ASD) is a lifetime multifaceted neuropsychiatric disorder featured by a constellation of early-appearing social communication and interaction deficits with repetitive sensory-motor behaviours; it is also characterized by a strong prevalence of males (American Psychiatric Association, 2013; Lord et al., 2022). ASD prevalence is continuously increasing with current estimations of 1/57 children, only partly attributable to a higher public awareness and better diagnostic criteria, which generates a substantial burden on public health care (Lord et al., 2018, 2022). Three severity dimensions (level 1, 2, and 3) were added into the Diagnostic and Statistical Manual of Mental Disorders, Fifth Edition (DSM-5) to specify patient’s needs and social functioning (American Psychiatric Association, 2013). It is common (about 70%) for children with ASD to have other neurodevelopmental disorders that may have similar dysregulated neurobiological pathways (Lord et al., 2022), mostly Intellectual Disability (ID; 40%), Attention Deficit Hyperactivity-impulsivity Disorder (ADHD 30%) (Lai et al., 2019; Carta et al., 2020), and Epilepsy (ranging 8 to 30%) (Lai et al., 2014), along with sleep disorders (i.e., insomnia, ranging 15%–80%) (Lai et al., 2019). The etiology of ASD is highly complex and involves interacting networks between genetic and environmental risk factors (Sanders et al., 2015; Kim et al., 2019; Willsey et al., 2022). Studies have associated over a hundred among genetic loci and prenatal environmental factors (stress, undernutrition, drugs exposure, and maternal immune activation), all contributing to increase the ASD risk in offspring (Betancur, 2011; Doi et al., 2022; Hirota and King, 2023). In recent years, the early prenatal immune-mediated hypothesis has gained credibility due to microglia activation in brain autoptic studies of individuals with ASD (reviewed in Sotgiu et al., 2020). Other neuroanatomical differences have been observed in the brains of individuals with ASD in their first years of life, such as brain volume overgrowth, cortical thickness, and increased volume of perivascular spaces (PVS), also named as Virchow-Robin spaces (Ecker et al., 2012). These spaces are located around penetrating arteries and are surrounded by astrocytic vascular endfeets that express aquaporin-4 (AQP4). AQP-4 water channels permit cerebrospinal fluid (CSF) flow into the brain through arterial PVS and then into the interstitial fluid. This mixed fluid is then carried toward venous PVS to reach meningeal lymphatic drainage and cervical lymph nodes (Iliff et al., 2012; Plog and Nedergaard, 2018; Taoka and Naganawa, 2020). This network, called the glymphatic system (GS), is central to brain homeostasis for fluid transportation and waste removal (Plog and Nedergaard, 2018; Troili et al., 2020). There is growing interest associating GS and PVS with neurological disorders, as these changes to GS could cause harmful protein build-up (Liu et al., 2020; Troili et al., 2020; Barisano et al., 2022). Some studies suggest that individuals with ASD tend to have increased levels of Aβ (Wegiel et al., 2012; Westmark, 2013; Westmark et al., 2016). Furthermore, the level of extra-axial CSF in the subarachnoid space remains abnormally high even at 3 years of age, whereas it increases from birth to 7 months and decreases between 12 to 24 months (benign external hydrocephalus) in typical development (Shen et al., 2013; Shen, 2018). Occasionally, macrocephaly caused by an excess extra-axial CSF volume may not be benign, rather suggesting a putative link between abnormal CSF circulation and the development of autism (Shen et al., 2017; Dinstein and Shelef, 2018). Being the subarachnoid space directly linked to the PVS, it is not uncommon to observe an enlargement of PVS in ASD population. A pioneering MRI study demonstrated brain PVS dilation in 7/16 ASD patients (Taber et al., 2004). Later, PVS enlargements were confirmed only in ASD with co-occurring developmental delay (Zeegers et al., 2006) and only in 20% of non-syndromic ASD patients (Boddaert et al., 2009). These tenuous associations were ascribable to the fallacy of visual assessment of visible and large-caliber PVS mainly in regions of interest. A recent study with a new diffusion tensor imaging along the perivascular spaces (DTI-ALPS) technique demonstrated that ASD patients can have a significant GS dysfunction, which is strictly related to age (Li et al., 2022). Other innovative techniques (Barisano et al., 2022) can improve the quantification of PVS and enable new clinical investigations. A novel MRI processing approach, with automated quantification of the combination of T1- and T2-weighted images and the filtering of non-structured noise, has been recently developed (Sepehrband et al., 2019) which strongly improves the contrast-ratio between PVS and the surrounding tissue, thus facilitating PVS mapping and quantification. Here, by using this innovative method, we conducted, for the first time, a single-center case–control study on PVS in children with ASD and with other neuropsychiatric disorders. Based on aforementioned studies, we hypothesized that an imbalance between the production and drainage of CSF in autism may result in an enlargement of PVS. Furthermore, individuals with ASD often experience sleep issues which, in principle, can contribute to worsen this imbalance (Xie et al., 2013).

## 2. Materials and methods

### 2.1. Participants

This study was approved by the Ethics Committee of Azienda Ospedaliero-Universitaria, Cagliari, Italy (PROT. PG/2023/5144), and all procedures were performed in accordance with the relevant guidelines and regulations. A written informed consent was obtained from parents or legal guardians of all young participants. Clinical files of children with ASD and with neuropsychiatric disorders other than autism (NP/Non-ASD group) have been retrospectively and consecutively collected at the Child Neuropsychiatry Unit, University Hospital, Sassari. To reduce the complexity of the population under study, we only included patients living and born in Sardinia between January 1st, 2010 and December 31st, 2021. To be eligible, all children, with an age ranged between 2 and 7 years, must have been previously subjected to brain MRI, in line with guidelines for assessment of comorbidity conditions in ASD (NICE—National Institute for Health and Clinical Excellence, n.d.). MRI investigations are not mandatory for individuals with ASD, but professionals in Italy, as in other countries, frequently use them as part of the diagnostic process. However, it is important to mention that MRI investigation may be necessary in children with ASD who have abnormal neurological examination, macrocephaly, dysmorphisms, headache, convulsions or EEG abnormalities. Indeed, their MRI scans reveal a greater occurrence of pathological findings (Rochat et al., 2020).

Besides, clinical files had to contain cognitive and behavioral assessments along with a complete medical review. Based on this, 81 patients with ASD and 114 patients of the NP/Non-ASD group were recruited. Out of the ASD group, 9 children were excluded due to incomplete medical records, 4 due to poor MRI quality, and 3 due to missing MRI plane data. Out of the NP/Non-ASD group, 43 patients were excluded for several reasons: 21 had incomplete medical records, 8 missed MRI sequences, 6 had missing data on MRI planes, and 8 had inadequate quality of MRI. Thereafter, 65 ASD (83%) and 71 NP/Non-ASD (65%) individuals were included, owing to the following exclusion criteria: inadequate quality of MR imaging, missing data on MRI planes and missing MRI sequences, incomplete medical records, MRI demonstration of massive malformations or tumors, massive stroke and/or hemorrhage.

### 2.2. Clinical methods

Diagnoses were supported by parent interviews, direct child assessment, review of available parent (and teacher for school-age children) questionnaires, as well as available prior records. At least two evaluators were involved in the diagnostic assessments, including licensed psychologists and/or supervised medical residents in child and adolescent neuropsychiatric, post- and pre-doctoral fellows. One evaluator conducted the parent structured interview with parents for the anamnestic history as well as earlier history and current use of psychoactive medications for the ADHD. Another evaluator administered and scored the Autism Diagnostic Observation Schedule, second edition (ADOS-2).

After these assessments, child and parent evaluators shared their observations, discussed their clinical impressions, and reviewed all available clinical information. To further characterize the sample, additional parent-report measures, such as the Child Behavior Checklist (CBCL; Achenbach, 1999) the Vineland Adaptive Behavior Scale (VABS; Sparrow et al., 1984) or the Adaptive Behavior Assessment System—Second Edition (ABAS-II; Harrison and Oakland, 2015) survey interviews were collected.

The diagnostic assessment was conducted by at least one licensed neuropsychiatrician, or at least one supervised resident medical doctor, and consisted of parent interviews using the Autism Diagnostic Interview—Revised (ADI-R; Rutter et al., 2003), child testing and observations using the ADOS-2. Additional parent measures were also collected including the CBCL and the ABAS among others. For all studies, ADOS-2 and ADI-R were administered and scored during clinical assessment by clinically trained staff currently in training for research reliability. For this purpose, consensus ADOS-2 scoring was obtained with a research-reliable specialist. Based on ADOS and ADI scores, clinicians detected the three levels of patient’s rehabilitation needs and performed a formal diagnosis for ASD in line with the DSM-5 criteria.

Cognitive abilities were assessed using the Wechsler Intelligence Scale for Children (WISC-IV; Wechsler, 2004) and the Wechsler preschool and primary scale of intelligence-III (WPPSI-III; Wechsler, 2002). LEITER International Performance Scale—revised (LEITER-R; Roid et al., 2013), or Raven’s Coloured Progressive (CPM) were selected based on the participant age and cognitive abilities. The Standard Progressive Matrices (SPM) (Raven, 1998) or the Griffith’s Mental Development Scale-Extended Revised (GMDS-ER; Luiz et al., 2006) were used when the child was likely to fail to complete any of the other cognitive scales because of the absence of language or his/her reduced attention resources. Some patients in the sample were not sufficiently collaborative and standardized tests results were considered invalid.

Detailed objective evaluation (to highlight dysmorphic traits), neurological examination (e.g., motor dyspraxia, pyramidal or extrapyramidal signs), clinical course (e.g., regression over neurodevelopmental phases) and anamnestic information (e.g., family history of ASD, other neurodevelopmental disorders, autoimmune diseases) were used to identify children with high risk for genetic mutations. Of these, those children with confirmed alterations on these clinical and genetic parameters were defined as possible syndromic ASD type. Based on these clinical selection criteria, children with high risk for syndromic ASD were screened for chromosomal heritable or *de novo* mutations, including micro- as well as macro-deletions/insertions. In detail, blood samples were collected to evaluate standard karyotype, FRAXA gene and array-CGH. In those with a previous specific familial history for chromosomal mutations, a FISH of the specific gene was requested. In our sample, we identify a total of 8 alterations: three children with heterozygous deletion at chromosome 22q11.2 on chromosomal microarray; one with 47XXY, one with 49XXXY and one with Down’s syndrome at the standard varitype analyses. Finally, two kids were found with Fragile-X syndrome.

In sum, the following clinical variables were considered for the eligible children with ASD in our study:

- ASD type (essential, complex/syndromic).
- ASD diagnosis confirmed by the Autism Diagnostic Observation Schedule (ADOS-2; Lord et al., 2012).
- ASD severity level (1, 2, and 3; American Psychiatric Association, 2013).
- Composite intelligence quotient (IQ) of the Wechsler Preschool and Primary Scale of Intelligence-III (WIPPSI-3) or Wechsler Intelligence Scale for Children (WISC-IV) scales (normal >85; borderline: 84–71; mild ID: 55–70; moderate ID: 40–55; severe ID<40).
- Griffiths Mental Development Scales (GMDS) to measure the rate of development of young infants.
- Comorbidities: ADHD, ID, Epilepsy, Sleep disorder (insomnia).
- Genetic testing.
- MRI standard report (increased PVS, abnormal CSF circulation, malformation, normal) (Table 1).

**Table 1:** Demographic, clinical and descriptive MRI analyses of the total cohort, stratified between NP/Non-ASD and autism cases.

Patients		Total cohort (*n* = 136)	NP/Non-ASD (*n* = 72)	ASD (*n* = 64)	*p*
Median (IQR) age, years		4 (2–6)	4 (2–7)	3 (2–5)	0.06
Females, *n* (%)		53 (39.0)	32 (44.4)	21 (32.8)	0.17
Complex autism (%)		–	–	54 (84.4)	–
ASD Severity *n* (%)	Mild 1	–	–	10 (15.6)	–
	Moderate 2	–	–	18 (28.1)	
	Severe 3	–	–	36 (56.3)	
IQ levels *n* (%)	Normal	19 (37.3)	11 (52.4)	8 (26.7)	0.34
	Borderline	16 (31.4)	6 (28.6)	10 (33.3)	
	Mild	7 (13.7)	1 (4.8)	6 (20.0)	
	Moderate	6 (11.8)	2 (9.5)	4 (13.3)	
	Severe	3 (5.9)	1 (4.8)	2 (6.7)	
ADHD, *n* (%)		13 (9.6)	3 (4.2)	10 (15.6)	**0.04**
Sleep disorders, *n* (%)		24 (17.7)	0 (0.0)	24 (37.5)	**<0.0001**
Epilepsy, *n* (%)		17 (12.5)	8 (11.1)	9 (14.1)	0.60
MRI findings					
PVS enlargement, *n* (%)		7 (5.1)	3 (4.2)	4 (6.3)	0.71
Abnormal size of CSF spaces, *n* (%)*		17 (12.5)	13 (18.1)	4 (6.3)	**0.04**
Gliosis, *n* (%)		27 (19.9)	14 (19.4)	13 (20.3)	0.90
Malformation, *n* (%)**		11 (8.1)	8 (11.1)	3 (4.7)	0.22
Normal, *n* (%)		62 (45.6)	31 (43.1)	31 (48.4)	0.53
Median (IQR) WM-PVS volume (n. of voxel)		545 (334.5–902.5)	532.5 (312.0–735.5)	621.5 (374–1,061)	0.07
WM-PVS grade, *n* (%)	1 (1–10)	6 (4.4)	5 (6.9)	1 (1.6)	0.10
	2 (11–20)	32 (23.5)	15 (20.8)	17 (26.6)	
	3 (21–40)	57 (41.9)	35 (48.6)	22 (34.4)	
	4 (>40)	41 (30.2)	17 (23.6)	24 (37.5)	

*Benign enlargement or restriction of ventricular size; benign enlargement of subarachnoid space; idiopathic aqueduct stenosis, megacisterna magna, Chiari I with or without syringomyelia. **Macrocephaly, microcephaly, cerebellar atrophy, abnormal septum pellucidum, abnormal corpus callosum, abnormal neuronal migration, abnormal cortical organization. Significant *p* values are in bold.

In the NP/Non-ASD group, ADHD diagnosis was based on developmental history and extensive clinical examination and further supported by the ADHD parent’s rated scale by Conners—CPRS (Conners et al., 1998). Epilepsy was diagnosed or excluded with wake and sleep electroencephalographic patterns along with patient’s history; sleep disorders were diagnosed with parent-report sleep screening instrument designed, the Children’s Sleep Habits Questionnaire (CSHQ) and wake and sleep electroencephalographic features (Owens et al., 2000). The adaptive and the cognitive profiles in the NP/Non-ASD group participants were evaluated throughout the same instruments used to test intellectual abilities in the ASD children. The following clinical variables were considered for children of the NP/Non-ASD group:

- Diagnosis other than ASD.
- Comorbidities: ADHD, ID, Epilepsy, Sleep disorder (insomnia).
- Composite IQ (as for ASD group).
- Griffiths Mental Development Scale.
- Genetic testing (when appropriate).
- MRI standard report (as above; Table 1).

### 2.3. MRI processing

**Figure 1:**
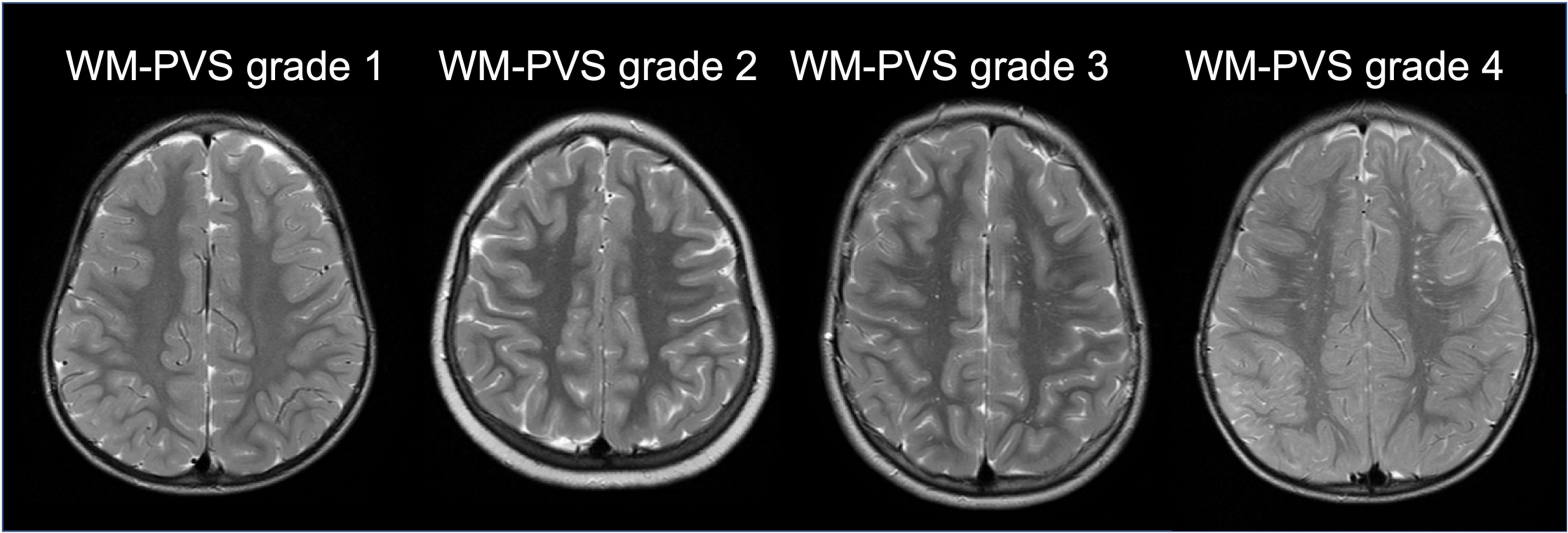
WM-PVS grades. Representative cases of the four WM-PVS grades observed in our cohort. WM-PVS on T2-weighted axial images appear as hyperintense structures in the white matter, linear or punctate depending on whether their course is parallel or perpendicular to the axial acquisition plane, respectively.

Standard MRI acquisition was performed, in all individuals, by using the same 1.5 Tesla Philips MRI scanner at the University Hospital in Sassari. The following sequences and planes were considered: T1-weighted spin-echo axial (TR: 357–895 ms; TE: 10–15 ms; flip angle: 69) and T2-weighted turbo spin-echo axial (TR: 4,000–7,000 ms; TE: 100 ms; flip angle: 90;) imaging with a spatial resolution of 0.8 × 0.8 × 4.4 mm^3^ and 0.4 × 0.4 × 4.4 mm^3^, respectively. One senior neuroradiologist (PC with 13 years of experience), blinded to clinical information, performed the MRI qualitative analysis. Special attention was given to evaluating shape/size of perivascular and subarachnoid spaces. Lateral ventricles were defined enlarged when the largest diameter perpendicular to the midline measured in axial slices on the frontal horn, body and/or the atrium was above 10 mm, whereas PVS were defined enlarged when there were at least 3 PVS whose diameter perpendicular to the PVS’ main axis was above 3 mm (Boddaert et al., 2009). Gliosis was identified in T2 sequences as areas with a higher signal intensity compared with the surrounding white matter. Other MRI abnormalities, such as mega cisterna magna, idiopathic aqueduct stenosis, Chiari I with or without syringomyelia were considered, as they may indicate abnormal CSF circulation. Cerebellar atrophy, abnormal septum pellucidum, abnormal corpus callosum, neuronal migration (cortical ectopias) and cortical organization (lissencephaly or focal cortical dysplasia) were also taken into account. These classifications were made qualitatively based on the overall impression of the neuroradiologist. Macro/microcephaly was assessed clinically based on head circumference. Spatial resolution, MRI planes and sequences of the two datasets (ADS vs. NP/Non-ASD) were comparable. Subsequent MRI processing was conducted using a semi-automated quantification algorithm for PVS. A multi-modal approach enhanced the PVS visibility by combining T1 and T2 images that were adaptively filtered to remove non-structural high frequency spatial noise (Sepehrband et al., 2019; Barisano et al., 2022). A white matter mask was generated with a fast and sequence-adaptive whole-brain segmentation algorithm applied on T1 images (Puonti et al., 2016), and was subsequently eroded by 1 voxel. T2 images were then rigidly transformed to the T1 space. White matter intensity was uniformized across space using the default values of the 3dUnifize function in AFNI (Cox, 1996). In the eroded white matter mask applied on the T2-weighted image, we calculated voxel-wise the average intensity difference between the voxel and its surrounding voxels. The intensity difference maps were reviewed blind to clinical status to the clinical and demographic information, and an intensity difference higher than 60 was used to define a voxel as a white matter PVS (WM-PVS) voxel. Since the slice thickness was larger than the PVS thickness, the number of voxels rather than the mm^3^ was the adopted measurement unit for the volume of white matter and PVS. Also, as the PVS volume is highly correlated with the WM volume (Barisano et al., 2021), the PVS volume value was normalized using the amount of white matter for each individual. Since the majority of PVS studies in the literature report only the visual scores of the MRI-visible PVS (Barisano et al., 2022), in our study we included a qualitative estimate of the extent of the PVS burden. Therefore, the number of WM-PVS voxel clusters was calculated. WM-PVS voxel clusters were identified automatically in each axial slice within the eroded white matter mask: PVS voxels were considered part of the same cluster if their edges or corners touch. Finally, we recorded for each subject the highest number of WM-PVS visible in a single axial slice, and adapted a validated 5-point scale (MacLullich, 2004) to define the following WM-PVS grades (Figure 1):

- WM-PVS Grade 0: denoted 0 PVS.
- WM-PVS Grade 1: denoted for 1 to 10 PVS.
- WM-PVS Grade 2: for 11 to 20 PVS.
- WM-PVS Grade 3: for 21 to 40 PVS.
- WM-PVS Grade 4: for more than 40 PVS.

### 2.4. Statistical analysis

Sample characteristics were summarized with medians and interquartile ranges (IQR) for quantitative variables, and with absolute and relative (percentages) frequencies for qualitative ones. Shapiro–Wilk test was used to assess normality of data. Comparison of qualitative variables was performed with Chi Square or Fisher exact test, whereas Mann–Whitney or Kruskal-Wallis test were used for the comparison of quantitative variables between two or more groups, respectively. Spearman’s correlation was performed to assess the relationship between the different MRI measurements. Statistical significance level was set at *p* < 0.05. STATA software version 17 (StataCorp, TX) was used to perform all statistical computations.

## 3. Results

Table 1 reported detailed demographic and clinical information between ASD cohort and NP/Non-ASD group. A total of 136 patients (61% males) were evaluated; of these, 72 (55.6% males) were cNP/Non-ASD individuals and 64 (67.2% males) children with ASD. The median age in the total cohort was 4 years (IQR: 2–6), 4 years in the NP/Non-ASD and 3 in the ASD group. Children with ASD were diagnosed with complex ASD in 84.4%, mostly with level 3 (severe autism 56.3%). Intellectual disability (from mild to severe) was found in 40% ASD and 19% NP/Non-ASD. ADHD and sleep disorders were significantly more represented within the ASD group (15.6 and 37.5%; *p* = 0.04 and < 0.0001, respectively). On the contrary, the frequency of epilepsy was equally distributed among the two groups (Table 1).

Descriptive standard MRI data indicated a significant impairment of CSF circulation within the NP/Non-ASD compared to ASD (*p* = 0.04; Table 1).

WM-PVS volume was directly related with the WM-PVS grade (Supplementary Figure S1). Median WM-PVS volume increases as WM-PVS grade increases, from 161 (IQR: 115–228) voxels in grade 1 to 1,114 (IQR: 949–1,422) voxels in grade 4.

Table 2A shows the difference of median WM-PVS volume and frequencies of WM-PVS grade according to sex in the whole cohort (ASD plus NP/Non-ASD). When males and females with ASD are included together, WM-PVS grade and WM-PVS volume do not significantly differ between the ASD group and the control group overall. We found, instead, that males had higher WM-PVS volume compared to females (*p* = 0.01).

**Table 2:** Comparison between PVS-WM volume and WM-PVS visual grades with (a) the sex of the whole cohort, (b) NP-Non-ASD versus ASD males, (c) NP-Non-ASD versus ASD females, (d) autism cases < and ≥ 4 years of age, and (e) according with ASD severity.

		(a) Whole cohort		(b) Male cohort		(c) Female cohort		(d) Age of ASD cases		(e) Severity of ASD		
Groups (n. of patients)		All males (83)	All females (53)	Male NP-Non-ASD (40)	Male ASD (43)	Female NP-Non-ASD (32)	Female ASD (n = 21)	<4 years (35)	≥4 years (29)	Mild level 1 (10)	Moderate level 2 (18)	Severe level 3 (36)
Median (IQR) WM-PVS volume (n. of voxel)		596* (361–1,099)	425 (306–701)	545 (353–931.5)	705 (392–1,196)	415 (267–660.5)	425 (337–730)	795** (431–1,252)	422 (324–657)	488.5 (332–657)	417 (294–855)	751.5 (434–1,155)
WM-PVS grade *n* (%)	1 (1–10)	3 (3.6)	3 (5.7)	3 (7.5)	0 (0.0)	2 (6.3)	1 (4.8)	1 (2.9)	0 (0.0)	0 (0.0)	1 (5.6)	0 (0.0)
	2 (11–20)	19 (22.9)	13 (24.5)	7 (17.5)	12 (27.9)	8 (25.0)	5 (23.8)	6 (17.1)	11 (37.9)	3 (30.0)	6 (33.3)	8 (22.2)
	3 (21–40)	35 (42.2)	22 (41.5)	22 (55.0)	13 (30.2)	13 (40.6)	9 (42.9)	10 (28.6)	12 (41.4)	5 (50.0)	6 (33.3)	11 (30.6)
	4 (>40)	26 (31.3)	15 (28.3)	8 (20.0)	18* (41.9)	9 (28.1)	6 (28.6)	18*** (51.4)	6 (20.7)	2 (20.0)	5 (27.8)	17 (47.2)

Significant results are indicated with asterisks. WM-PVS grade 4 showed a progressive increase in frequency in relation to the progressive severity of ASD (20.0%, 27.8% and 47.2% in the mild, moderate, and severe level, respectively). *p = 0.01; **p = 0.006; ***p = 0.03.

Considering the cohort of only males, children with ASD also showed a higher median WM-PVS volume (705, IQR 392–1,196) compared to males of the NP/Non-ASD group (545, IQR 353–931.5; Table 2B) and a significant difference in the WM-PVS grade, being the extreme grade (grade 4) more represented in ASD than in NP/Non-ASD males. On the contrary, no difference was found in WM-PVS volume and WM-PVS grade between female ASD and female NP/Non-ASD (Table 2C).

**Figure 2:**
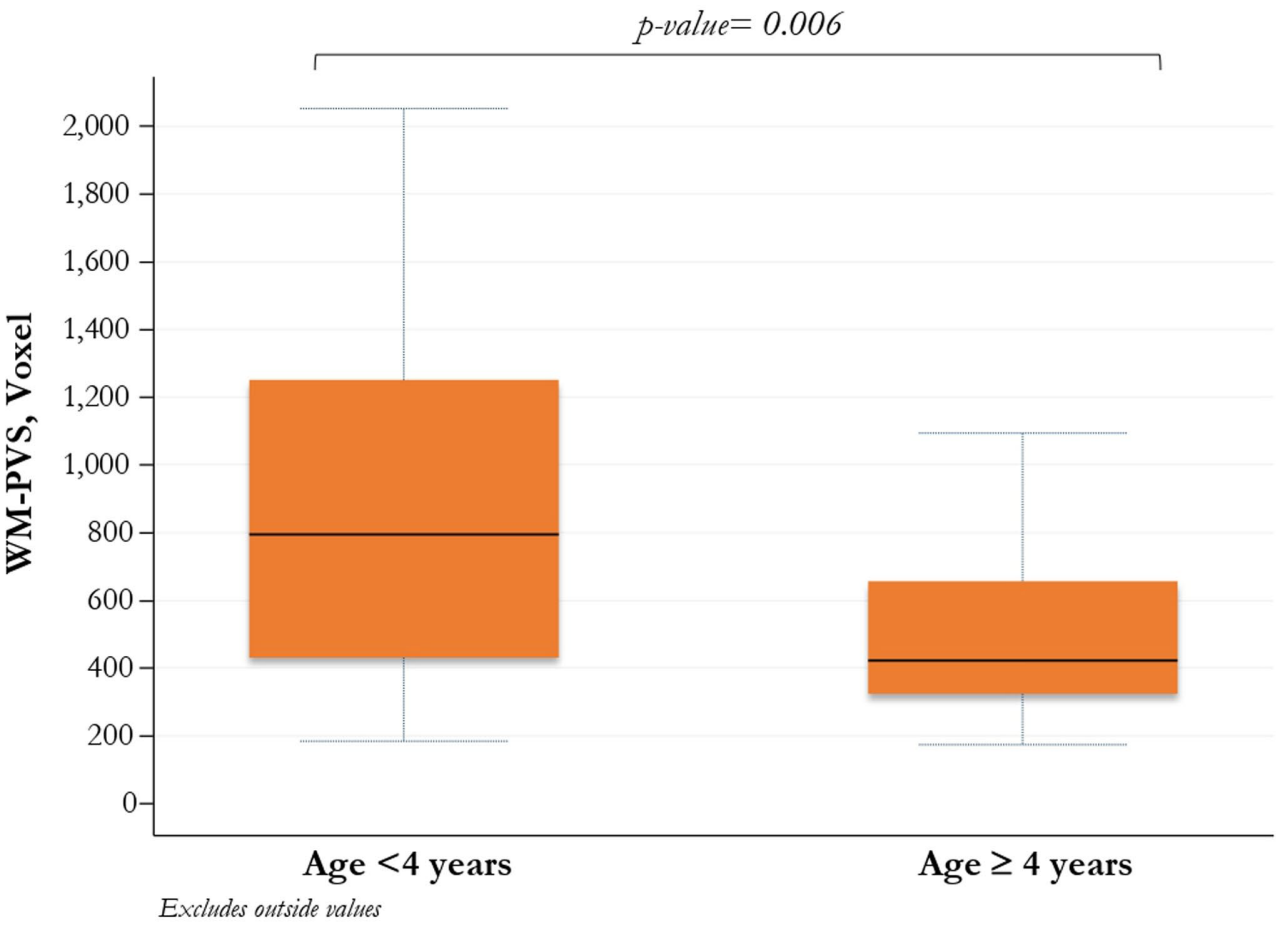
Comparison between WM-PVS volume and ASD patients (<4 years/≥ 4 years). PVS_WM enlargement is significantly more present at a younger age in ASD patients.

Stratifying children with ASD by age < and ≥ 4 years, both median WM-PVS volume and frequency of WM-PVS grade 4 were higher in younger ASD patients compared to those with 4 years or more (795, IQR: 431–1,252 vs. 422, IQR; 324–657; *p* = 0.006, and 18, 51.4% vs. 6, 20.7%; *p* = 0.03, respectively) (Table 2D and Figure 2).

**Figure 3:**
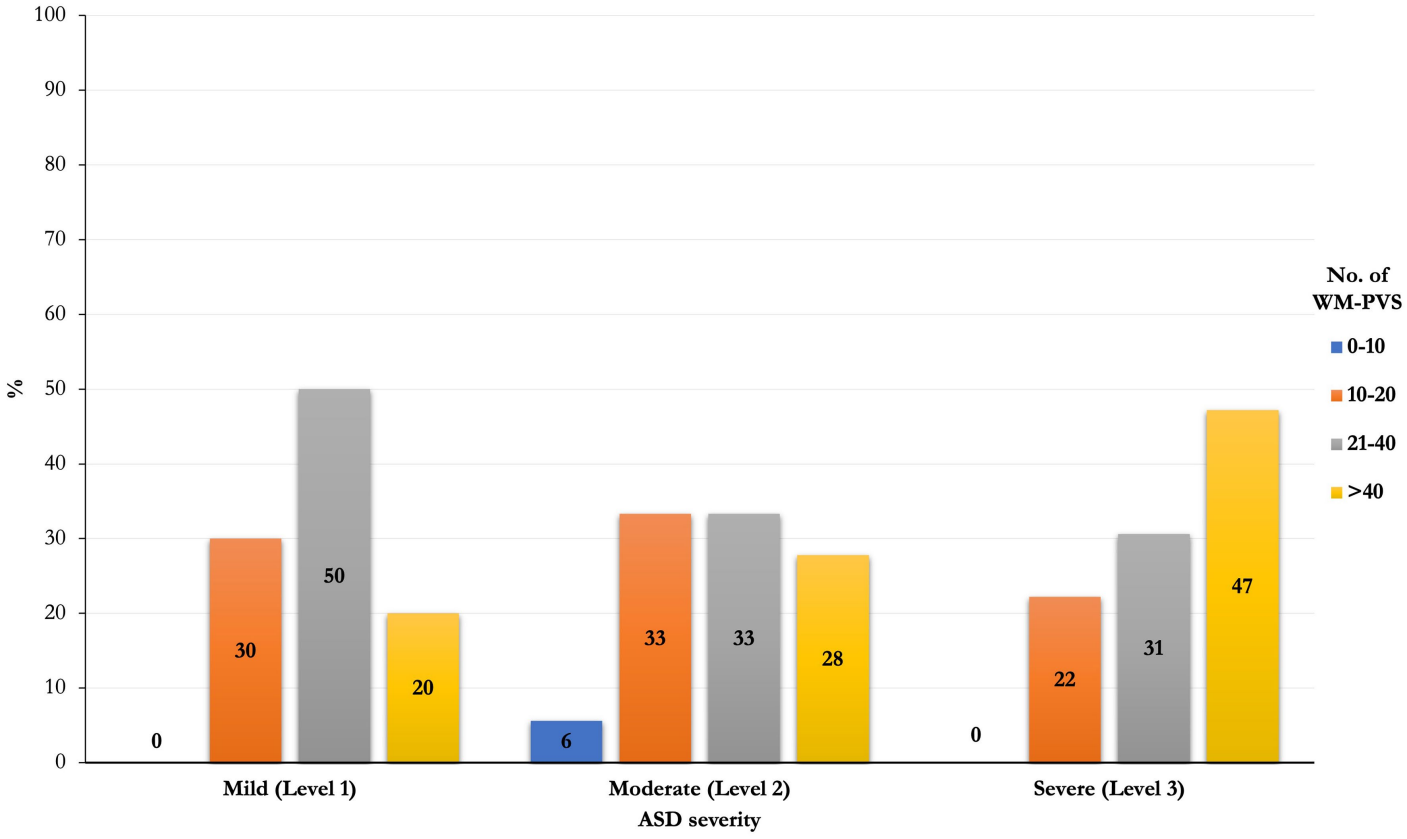
Frequencies distribution of PVS class between severity of ASD. An increasing prevalence of the number of WM-PVS more than 40 emerged as the ASD severity worsened (level 1 to level 3): only 20% of ASD patients with mild severity (level 1) had WM-PVS Grade 4, while 47% of level 3 severity ASD patients had WM-PVS Grade 4. This trend is not significant (*p* = 0.22).

Although not significantly (*p* = 0.22). an increasing prevalence, of the number of WM-PVS more than 40 emerged as the ASD severity worsened: only 20% of ASD patients with mild severity (level 1) had WM-PVS Grade 4, while 47% of level 3 severity ASD patients had WM-PVS Grade 4 (Table 2E and Figure 3). Among the ASD-associated comorbidities (ADHD, Epilepsy, ID, Sleep disorders), the highest WM-PVS grade was directly related to insomnia but not to other comorbid disorders, although rating of sleep co-occurring condition in both cohorts was lower compared to the expected rate from literature.

## 4. Discussion

Apart from the rare monogenic ASD forms, ASD etiology and the heterogeneity of its symptoms constellation involve a complex interaction of genes, environment, epigenetic and immunological factors. None of these factors alone are enough to fully explain the causes of ASD (Kim et al., 2019; Lord et al., 2020; Trost et al., 2022). Consequently, the vast majority of autistic disorders remains of undetermined origin and attempts to discover common risk factors for all/most ASD forms seem yet destined to fail. In general, core ASD features, age at onset and subsequent clinical trajectories vary substantially from one individual to another, making it necessary to define distinct ASD subtypes (Lord et al., 2018). ASD forms can show heterogeneity at their onset being ASD occurrence classified of either an “early” or “regressive” type. In the early-onset ASD, children present with early problems in behaviour and interaction, while in the regressive pattern the loss of communication is manifested later, conventionally after having learned 5 words and used them for at least 3 months (Zwaigenbaum, 2019). Male children with regressive ASD onset can have abnormal, though transient, age-dependent brain enlargement on MRI (Nordahl et al., 2011), associated with an excess of peripheral pro-inflammatory cytokines (Ashwood et al., 2011). Interestingly, children later diagnosed with autism, have shown, on MRI, a transient excess volume of extra-axial CSF (EA-CSF) in the subarachnoid space, which did not correlate to the reduced brain parenchymal size (Shen et al., 2018). Here the CSF, that circulates in two compartments divided by a lymphatic-like membrane called SLYM (Møllgård et al., 2023), is in direct communication with PVS. PVS, typically <3 mm, are not seen on standard MRI sequences, but become visible when they are enlarged as a consequence of glymphatic flow reduction (Potter et al., 2015; Mestre et al., 2017).

In children, PVS dilation is not a common finding, with the exception of some rare genetic/metabolic diseases, such as mucopolysaccharidoses (Zafeiriou and Batzios, 2013). Some association has been initially reported in ASD (Taber et al., 2004; Zeegers et al., 2006; Boddaert et al., 2009) based on studies with traditional visual and subjective MRI measuring. A recent study with an innovative DTI-ALPS method has assessed an age-related GS dysfunction in a limited group of patients with ASD (Li et al., 2022). With our study we aimed at giving more insight into the relationship between ASD and WM-PVS by increasing the sample size (this is the largest study conducted so far) and by using a novel MRI-processing approach which ameliorates PVS mapping and quantification (Sepehrband et al., 2019; Barisano et al., 2022). Also, for the first time, we aimed at investigating the association between WM-PVS and ASD severity and associated comorbidities.

The first result of the study is that WM-PVS volume is significantly associated with the male sex. All males (both ASD and NP/Non-ASD) are distinguished by a significantly higher WM-PVS volume compared to females, in line with previous results (Zhu et al., 2011; Barisano et al., 2021). Additionally, the highest WM-PVS grade is more frequent in male ASD patients than NP/Non-ASD children, while no differences were found in females. Results are in line with the concept that male epidemiological preponderance in autism may rely on male-specific risk mechanisms and/or female-specific protective mechanisms (Floris et al., 2021). These mechanisms can act early during neurodevelopment although age and comorbidities strongly modulate the pattern of differences later in life (Lai et al., 2017).

In our study, frequency distribution of WM-PVS grades according to different severity levels of ASD shows an increasing trend, with 80% “mild” ASD patients with less than 40 WM-PVS (WM-PVS grade 0–3) in a single MRI section and 53% “severe” ASD patients with more than 40 visible WM-PVS (WM-PVS grade 4). Consistently, patients with ASD and insomnia tend to have higher WM-PVS volume compared to ASD patients without sleep issues, although this result was not statistically significant. Previous studies reported an association between higher PVS visibility on MRI and impaired sleep quality and efficiency (Berezuk et al., 2015; Aribisala et al., 2020) and obstructive sleep apnea (Song et al., 2017). In fact, sleep has been shown to be important for a proper functioning of the glymphatic system (Hablitz and Nedergaard, 2021).

Moreover, PVS dilation is significantly associated with a younger age in ASD patients (less than 4 years in our study). This finding is of particular importance as it corroborates the aforementioned age-related GS dysfunction in patients with ASD studied with DTI-ALPS MRI (Li et al., 2022) and the transient excess volume of EA-CSF in children who have been diagnosed as ASD later in their life (Shen et al., 2018).

Finally, we found no relation between WM-PVS and epilepsy or IQ. A recent study in a large sample of healthy young adults found no association between WM-PVS and cognitive function (Barisano et al., 2021), while in pediatric idiopathic generalized epilepsy and post-traumatic epilepsy MRI-visible PVS alterations have been described (Duncan et al., 2018; Liu et al., 2020). This disagreement is possibly due to the relatively low number of patients with epilepsy (17/136) in our study, which was not specifically focused on epilepsy. ADHD alone or in comorbidity seems also not to be related to PVS enlargement, possibly because ADHD is mediated by factors unrelated to immune-mediated or PVS-related mechanisms (Sotgiu et al., 2020; Carta et al., 2022).

While in previous studies the MRI-visible PVS were typically assessed through visual rating systems which provided a qualitative estimate of the PVS burden, current neuroimaging tools allow to perform segmentation of PVS on brain MRI. This advancement, coupled with improvements in quality and spatial resolution of the MRI data, increased the sensitivity in PVS detection on MRI and is leading to the exploration of novel quantitative and morphological features of MRI-visible PVS and their use as neuroimaging biomarker for neuropsychiatric disorders.

Our study does have limitations. Anamnestic data collection might have been impaired by the retrospective nature of the study. To overcome this problem, we used a very strict selection of the patients. The ADHD rates are generally considered to be up to 50% in school-aged autistic children. With the selection criteria given the age range of our participants may be undercounting an ADHD condition and, in principle, can affect results in our non-ASD cohort. Another limitation is the use of 2D MRI data, which prevented us to compute 3D maps of WM-PVS. However, this flaw was homogeneously distributed throughout the study groups. Also, we acknowledge that, being a hospital-based study, we may have selected for more severe and complex ASD cases and could not identify matched NP/Non-ASD children without neuropsychiatric disorder, which may have biased the final outcome. However, sex male is more represented in all neurodevelopmental disorders and, in line with this naturalistic representation, it could be seen as strength of our clinical study. On the other hand, a possible bias is related to attainment bias: the sample might be skewed to the level 3 of ASD, being underpowered to detect PVS in children with mild phenotype of ASD (level 1 and 2), overcalling the relationship between volume of PVS and clinical severity. Finally, the impact of ASD at this young age grouping (<4 years) may, in theory, affect cohort assignment, particularly in the youngest group. However, no significant differences emerged in comparison groups for the age assignment criteria (0–3 or 2–4 years).

With the given limitations, we found that, compared with NP/Non-ASD, ASD male, but not female, patients present more frequently a high number of WM-PVS (WM-PVS grade 4) on MRI. WM-PVS grade 4 was also more frequently observed in ASD patients younger than 4 years of age compared with older ASD patients. Additionally, we observed a trend for a higher frequency of WM-PVS grade 4 in patients with severe forms of autism compared with milder forms, and for higher WM-PVS volume in ASD patients with sleep disturbances compared with those without. Our study may shed new light on the alleged, though formerly weak, association between ASD and GS dysfunction.

Our results suggest that PVS dilation is a neuroimaging feature of ASD in male patients and is particularly evident in younger ASD patients (< 4 years of age), corroborating the findings of early mechanisms related to the transient excess volume of EA-CSF (and PVS dilation) acting during neurodevelopment, being modulated later in life (Lai et al., 2017; Shen et al., 2018; Li et al., 2022).

We believe that PVS could serve as a biomarker for identification of early and severe forms of autism and that the impairment of GS, resulting in the enlargement of PVS, could act as a cofactor which contribute to the worsening of the ASD clinical severity. Based on our findings, volumetric and morphometric MRI studies can be included in the clinical evaluation of the patient, particularly in children with severe ASD and syndromic ASD. Currently, we are unable to forecast potential therapeutic implications of our findings, which are planned in next studies.

## Data availability statement

The raw data supporting the conclusions of this article will be made available by the authors, without undue reservation.

## Ethics statement

The studies involving human participants were reviewed and approved by Ethics Committee of Azienda Ospedaliero-Universitaria, Cagliari, Italy (PROT. PG/2023/5144). Written informed consent to participate in this study was provided by the participants’ legal guardian/next of kin.

## Author contributions

MS, AL, GB, LS, VC, AM, PC, AC, and SS contributed to the study conception and design. Material preparation, data collection, and analysis were performed by GB, AL, LS, VC, and PC. Critical revision of the article for important intellectual content—AC and AM. The manuscript was written by AL, GB, MS, and SS. All authors contributed to the article and approved the submitted version.

## Funding

This work was supported by “BANDO FONDAZIONE DI SARDEGNA 2022 E 2023—PROGETTI DI RICERCA DI BASE DIPARTIMENTALI (D.R.16/2022)” grant. The funding sources were not involved in study design, collection, analysis, interpretation of data and in the writing of the report.

## Conflict of interest

The authors declare that the research was conducted in the absence of any commercial or financial relationships that could be construed as a potential conflict of interest.

## Publisher’s note

All claims expressed in this article are solely those of the authors and do not necessarily represent those of their affiliated organizations, or those of the publisher, the editors and the reviewers. Any product that may be evaluated in this article, or claim that may be made by its manufacturer, is not guaranteed or endorsed by the publisher.
