# Supplementary figures and images for: Brain perivascular spaces and autism: clinical and pathogenic implications from an innovative volumetric MRI study
Source: Front Neurosci. 2023 Jun 23;17:1205489. doi: 10.3389/fnins.2023.1205489 (PMC10328421; doi:10.3389/fnins.2023.1205489)

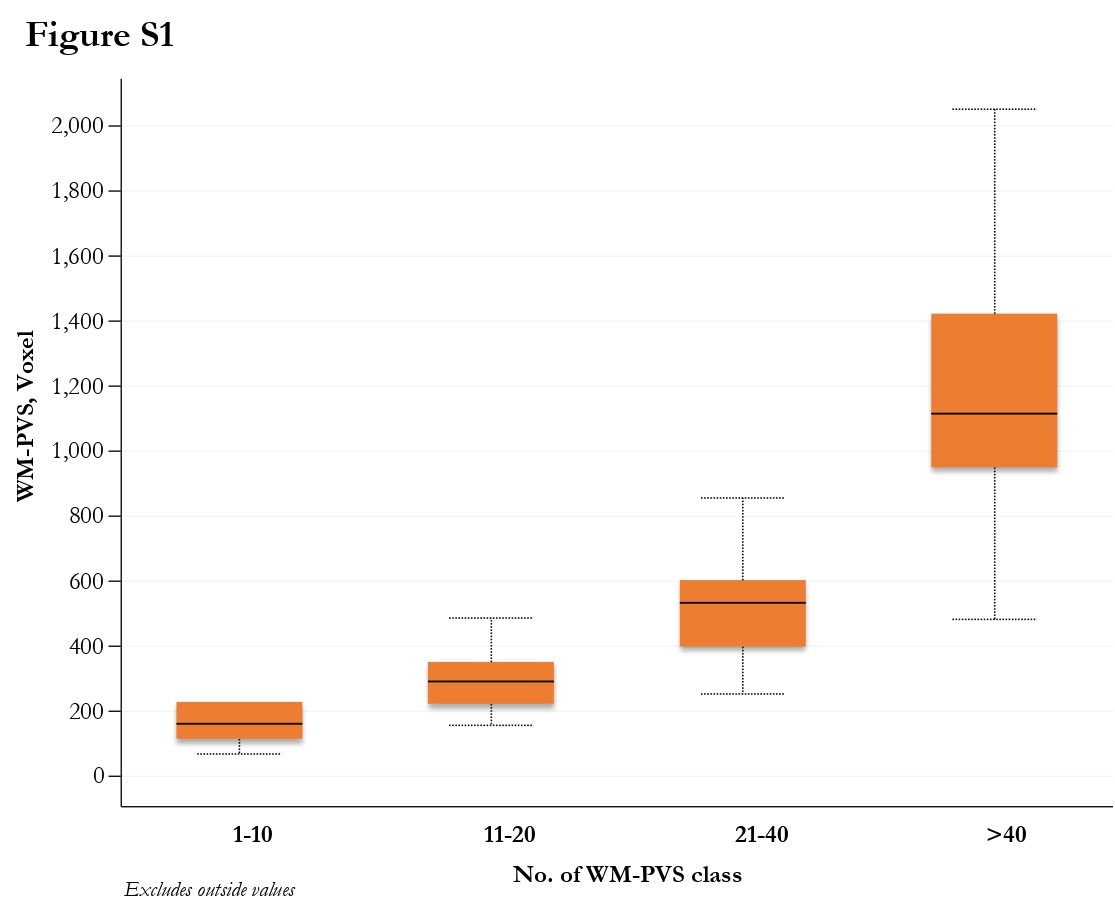

Supplement: Supplementary file 1 [file Image_1.JPEG]
